# Clinical Resolution of Plantar Warts Using the Needling Technique

**DOI:** 10.3390/diseases13020050

**Published:** 2025-02-07

**Authors:** María-Luisa Sobrín-Valbuena, Alberto Aldana-Caballero, Laura Martín-Casado, Inés Palomo-Fernández, Raquel Mayordomo, Félix Marcos-Tejedor

**Affiliations:** 1 Clini-K ^s.v^, 24402 Ponferrada, León, Spain; info@cliniksv.com; 2 Department of Nursing, Physiotherapy and Occupational Therapy, Faculty of Health Sciences, University of Castilla-La Mancha, 45600 Talavera de la Reina, Toledo, Spain; alberto.aldana@uclm.es (A.A.-C.); laura.martincasado@uclm.es (L.M.-C.); ines.palomo@uclm.es (I.P.-F.); 3 Department of Anatomy, Cellular Biology and Zoology, University of Extremadura, 10600 Plasencia, Caceres, Spain; 4 Department of Medical Sciences, Faculty of Health Sciences, University of Castilla-La Mancha, 45600 Talavera de la Reina, Toledo, Spain; felix.marcostejedor@uclm.es

**Keywords:** HPV, needling, plantar warts, punction, treatment

## Abstract

Background: Plantar warts, caused by the human papillomavirus (HPV), are a common skin condition characterized by painful lesions on the soles of the feet. These lesions can significantly impact skin appearance, quality of life, and, in severe cases, mobility. Traditional treatment methods, such as chemical cauterization or pharmaceutical therapies, are often painful and require multiple visits to achieve complete wart removal and skin regeneration. This study aims to assess the clinical effectiveness of the needling technique as an alternative treatment. This method involves repeatedly puncturing the lesion under local anesthesia or posterior tibial nerve block to trigger an immune response and promote wart clearance. Methods: A total of 26 patients underwent the needling procedure, which included puncturing the wart under local anesthesia, followed by wound dressing and topical application of iodopovidone to facilitate scab formation. Follow-up visits were scheduled until full wart resolution was observed. Analgesics were provided for moderate pain management when necessary. Results: After 30 days, a success rate of 57.7% was achieved. Patients reported mild pain, which subsided within a few days, and expressed high levels of satisfaction with the treatment outcome. Conclusions: The needling technique emerges as an effective alternative to chemical treatments, offering a notable wart clearance rate. Its use under local anesthesia enhances patient comfort and reduces treatment-associated anxiety compared to conventional therapies.

## 1. Introduction

Plantar warts are common lesions found in clinical practice, produced by an HPV (Human Papilloma Virus) infection, particularly types that affect the skin, such as HPV-1, HPV-27, and HPV-57 [1,2]. The annual incidence has been described to be up to 14%, and the condition affects mainly children and young adults, with slightly more women than men being affected and with special outcomes in immunocompromised patients [1,2,3,4].

The changes observed in the tissue include a disorganized granular layer with the presence of koilocytic cells and parakeratotic cells with nuclear inclusions in more superficial layers [5]. The clinical changes in the natural appearance of the tissue can play a key role, along with the symptoms associated with the lesions, in the decision to seek medical assistance to restore healthy skin.

Signs and symptoms include moderate to severe pain, which can lead to an inability to walk. Reactive hyperkeratosis covers the lesion, and vascular capillaries become visible when paring the skin, one of the key features used for visual diagnosis. Other diagnostic features include elevation, border erythema, or the presence of a callus [6]. The infection can manifest as simple or mosaic warts [4].

Infection can occur through direct exposure to the virus from an infected person. Autoinfection may also occur from an active lesion on the same individual. Additionally, indirect transmission has been described, particularly in high-traffic areas where people walk barefoot, such as swimming pools, as the virus can survive on surfaces for long periods [7].

There is a wide range of treatments available, most of which aim to destroy the lesional tissue, using different types of acids such as salicylic, nitric, trichloroacetic, or monochloroacetic acid, leading to epidermal lysis. Other pharmacological agents include retinoids, 5-fluorouracil, cimetidine, and bleomycin [4,8]. However, these therapeutic options have the limitation of affecting both healthy and lesional tissue, which can be problematic in cases of hypersensitivity. Alternatively, mechanical damage of the lesion can be achieved using other methods [8,9]. The Falknor’s needling technique, first described in 1969, has recently renewed interest. This method involves repeatedly inserting a needle into the lesion, inducing trauma that allegedly triggers an immune response to facilitate wart clearance. Unlike other treatments, it preserves the integrity of the surrounding skin, reducing aesthetic concerns for patients [10,11,12].

This study aims to report the outcomes of patients with plantar warts on the foot treated using the needling technique, providing further evidence of the effectiveness of this therapeutic approach for HPV plantar warts. Additionally, it explores the potential of this method to better preserve the surrounding healthy tissue and overall appearance of the skin.

## 2. Materials and Methods

### 2.1. Subjects

The recruitment was carried out based on the convenience of the subjects who attended the podiatry clinics Clini-K ^SV^ (Ponferrada, Spain) and the Podiatric Care Center of the University of Castilla-La Mancha (Talavera de la Reina, Spain). Signs and symptoms were assessed, and clinical diagnosis was given after visual inspection of the lesions [6]. Patients were excluded from the procedure if they had a previously diagnosed neuropathy or systemic disorder that might interfere. After being informed about the procedure, all subjects gave their approval and signed the informed clinical consent form. For the purposes of this study, all clinical information regarding the outcomes was analyzed retrospectively.

### 2.2. Preoperative Procedures

**Figure 1:**
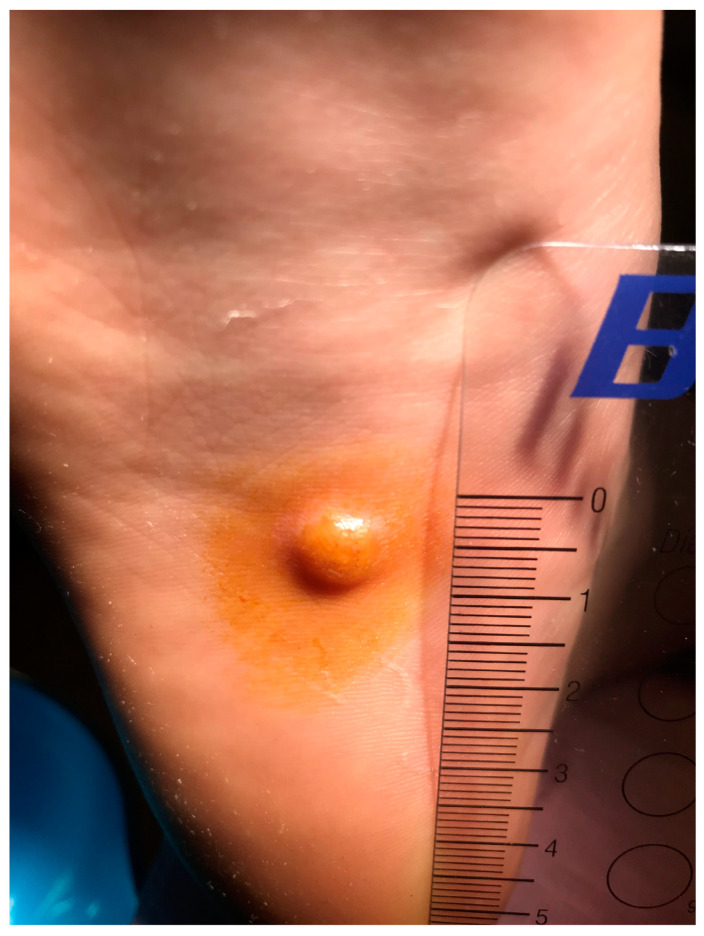
Lesion after preoperative procedures.

Cleaning of the entire foot was performed using surgical soap (20 mL 0.8% chlorhexidine gluconate), followed by iodine povidone being thoroughly applied. See Figure 1 for the starting point reference. The surface skin of the lesions was pared to expose it and to make the needling easier.

The anesthetic procedure was taken at this point using between 1 mL to 6 mL of mepivacaine 2%, which has a reasonable duration of action and a rapid onset [13], blocking the posterior tibial, metatarsal, or digital nerves, or, in milder cases, the perilesional injection was enough to numb the area prior to the treatment. The election of the anesthetic approach was based on the location of the lesion, severity, and pain threshold of the patients. In some severe cases, a complete blocking of the nerves of the ankle was be needed.

### 2.3. Needling Technique

A 25 G intramuscular needle was used for puncturing, starting from the outer borders of the lesion and moving toward the center. The needle was handled like a dart, and puncturing was performed until no resistance was felt from the tissue, ensuring adequate bleeding afterward.

**Figure 2:**
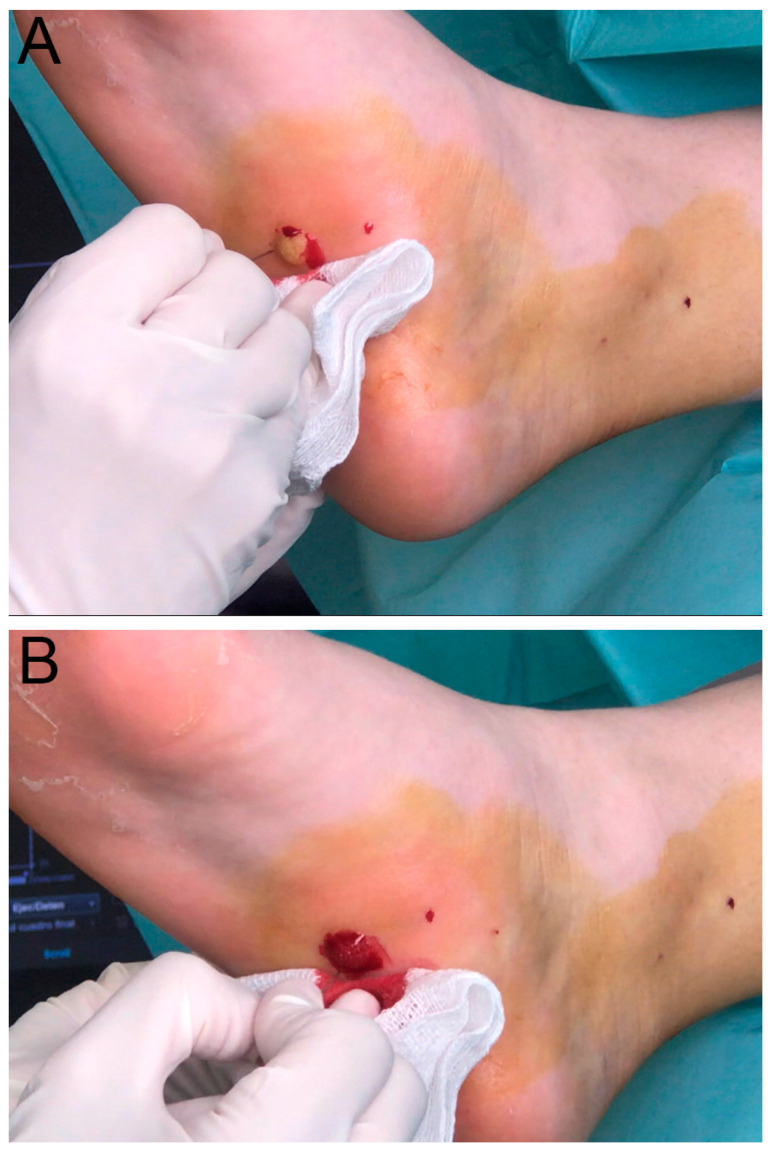
(**A**) Puncturing the lesion with the needle. (**B**) Bleeding of the multiple punctions to form the scab.

Punctures were repeated as many times as necessary to ensure complete coverage of the lesional tissue. For an 8 mm diameter lesion, up to 70 punctures might be required. Bleeding is a crucial step in the procedure until it naturally dries and forms a clot. This process is illustrated in Figure 2A,B.

Topical antiseptic was applied followed by a sterile dressing, elastic bandage, and padding to protect the area.

### 2.4. Postoperative Treatment

Dressings were kept properly during the first 12 h and iodopovidone was applied daily. Paracetamol 650 mg was prescribed when subjects described any type of postoperative pain or discomfort. Patients were followed on a weekly basis to monitor healing. A scab forms over the lesion and eventually falls off naturally around 30 days after the procedure, leaving healthy rosy skin underneath. In cases of an insufficient response to the treatment, another application or an alternative treatment could be considered to completely eliminate the wart.

### 2.5. Collecting of Data

The data collected for statistical analysis included demographic information (age, gender), lesion localization, time of evolution, presence or absence of hyperhidrosis, post-procedure pain (categorized as nonexistent, mild, moderate, or severe), and previous treatments, if applicable, in patients with recurrent lesions.

## 3. Results

### 3.1. Sample Characteristics

A total of 26 cases were treated, with a mean age of 24.04 ± 13.63. The cohort included 15 women and 11 men. The mean time of evolution of the lesions until the first visit was 32 ± 52.61 weeks.

One case had 35 lesions affecting different areas of the foot including the heel, interdigital space, and the top of the foot. Both feet were indistinctly affected by the infection.

In total, 19 cases were taking treatment for the first time, whilst 7 of them had been previously treated using nitric acid or folk remedies.

For further information about the cases refer to Table 1.

### 3.2. Anaesthesia and Pain Management

The anesthetic dose applied ranged from 1 to 6 mL, mean of 3.38 ± 1.34 mL. Analgesics were prescribed to 13 patients in order to reduce pain following the procedure.

### 3.3. Performance of the Technique

Patients did not need a further approach to the lesions in 22 of the cases. In total, 6 patients needed 1 or 2 applications of nitric acid afterward to successfully eliminate the lesion. The 30-day success rate was 57.7%, and the success rate after 60 days was 80.7%. Regeneration of the lesional tissue was a success in all the cases that resulted in clearance of the wart.

### 3.4. Statistical Analysis

Microsoft Excel v. 16.27 was used to keep track of the data of patients. SPSS v. 28 was used for the management of data and synthesis of results.

## 4. Discussion

HPV plantar warts do not have a treatment that specifically targets the infection, leaving health professionals with a wide range of options, as the main objective is to target the symptoms while eliminating the lesion. Most of these treatments have the intrinsic nature of being destructive to both healthy and lesional tissue, require several visits to apply, are painful, and have side effects [8,13]. In the present study, the needling technique proved to be more effective, with patients reporting no pain or only mild to moderate in nine cases. Furthermore, in 57.7% of the cases, a single needling procedure was followed enough by consecutive follow-ups to wash and heal the tissue until natural appearance was reinstated, and another 23% resolved in a subsequent intervention whilst most of the chemical burns or cryotherapy needed more applications [14].

It has been described that HPV warts may disappear spontaneously over the course of time, but the fact that plantar warts tend to include themselves into the skin in high-pressure bearing areas means that they cause intense pain and make patients seek assistance [3]. The needling technique has been reviewed in the last few years, without meeting agreement yet on its effectiveness [11,15,16]. Historically, since this technique was first documented in 1969, its effect has been suggested to rely on immunological mechanisms, activating an inflammatory response to the trauma. Although the literature lacks studies supporting this claim, it can be assumed to be true, considering the fact that the natural course of an HPV infection downregulates the immune system through the expression of E5–E7 proteins and maintains a low copy number in the basal layer to evade immune detection [10,11,12,17]. Since the infection naturally requires an immune response mediated by Langerhans cells and inflammatory cytokines [1], it is plausible that artificially damaging the lesional tissue with the needling technique could activate this response and contribute to viral clearance. This mechanism is similar to photodynamic therapy for plantar warts, which has been described as effective for a range of skin diseases by inducing cytokine production and stimulating the immune system [18]. However, further studies are still needed to confirm this effect.

In a recent clinical trial by Hashmi et al. [11], the technique was put into comparison with a control group. The results displayed that the only significance derived from the study was that patients under the needling technique treatment reported a more significant reduction in pain compared to those who underwent a simple removal of the superficial hyperkeratosis of the lesion. However, in this study, 57.7% (n = 15) of the patients achieved complete clearance of warts within 30 days of healing after treatment, with no recurrence reported. This percentage increased to 80.7% (n = 21) when including cases that required a longer time for complete resolution or a second intervention. Our results are in accordance with another randomized control trial by Cunningham et al. [15], which studied 37 patients, with a success rate for clearance of 65%, and a study by Skilton et al. [19] that described a rate of 50%, although this study was not a randomized control trial.

Pain is an important motive to make patients decide to treat their plantar warts, and it is related to how the treatment option is perceived by them. In the present study, paracetamol was prescribed as an analgesic drug for postoperative pain, and none of the patients reported further pain or discomfort after 1 month, which is a positive outcome to consider. Hashmi et al. [11] also reported a significant reduction in pain, and another study of 46 cases treated with the needling technique by Longhurst et al. [16] described a general reduction in pain and size of the warts.

Although our results are positive, more in-depth research on this subject is still needed, comparing different techniques or those in combination and considering the time needed for healing the tissue and the psycho-social implications on the patient’s quality of life.

A limitation of this study is the lack of long-term follow-up. Patients were advised to return to the clinic if they experienced any signs or symptoms suggestive of recurrence. However, none of them did. Although reliable data cannot be retrieved, it is reasonable to assume that they did not experience any recurrence after treatment, including the four cases that required additional treatment with nitric acid.

## 5. Conclusions

In conclusion, the needling technique proves to be a useful therapeutic option, particularly for patients who are apprehensive about chemical burns or sensitive to the products used. Additionally, topical anesthetics provide peace of mind, as most available treatments for plantar warts are associated with pain.

## Figures and Tables

**Table 1:** Sample characteristics, pain, and outcome of the lesions.

Type of Lesions		Localization			Hyperhidrosis		Pain			Complete Resolution
Simple	Mosaic	Metatarsal heads	Hallux	Heel	14		No	Mild	Moderate	15
17	8	8	8	6	Treated	Untreated	19	4	5	
					8	6				
TOTAL = 26 patients										

## Data Availability

No data available.
